# Cadmium Absorption in Various Genotypes of Rice under Cadmium Stress

**DOI:** 10.3390/ijms24098019

**Published:** 2023-04-28

**Authors:** Kaixuan Feng, Jiangxia Li, Yachun Yang, Zhong Li, Wenge Wu

**Affiliations:** 1 School of Resources and Environment, Anhui Agricultural University, Hefei 230031, China; 2 Rice Research Institute, Anhui Academy of Agricultural Sciences, Hefei 230031, China; 3 School of Resources and Environment, Anhui University, Hefei 230031, China; 4 College of Advanced Agricultural Sciences, Zhejiang A&F University, Hangzhou 311300, China

**Keywords:** rice organ, heavy metal stress, antioxidant enzymes, RT-qPCR, variety screening

## Abstract

Cadmium (Cd) is a kind of heavy metal. Cadmium pollution in paddy fields will accumulate a large amount of cadmium in rice, which will affect the growth and development of rice. In addition, long-term consumption of rice contaminated with Cd can harm human health. In this study, four rice varieties with high Cd accumulation (S4699, TLY619, JHY1586, QLY155) and four varieties with low Cd accumulation (YY4949, CYJ-7, G8YXSM, MXZ-2) were screened through field experiments for two consecutive years (2021 and 2022) and differences in antioxidant enzyme systems and expression of genes in their organs were analyzed. The total Cd content showed as follows: indica rice > japonica rice, high-Cd-accumulation variety > low-Cd-accumulation variety, and the total Cd content of each organ of rice showed root > stem > leaf > grain. The results of the antioxidant enzyme system showed that the contents of malondialdehyde (MAD), reduced glutathione (GSH), oxidized glutathione (GSSH), and peroxidase (POD) were positively correlated with the total Cd content in rice, and superoxide dismutase (SOD) showed the opposite performance in the leaves. There was no correlation between catalase (CAT) and Cd content, but CAT content decreased in leaves and grains and increased in roots and stems with increasing fertility. Based on this study, RT-qPCR was used to further validate the expression of Cd-uptake-related genes in different rice varieties. It was found that high expression of *OsHMA3*, *OsCCX2*, *OsNRAMP5*, and *OsHMA9* genes promoted Cd uptake and translocation in rice, especially in rice varieties with high Cd accumulation. The high expression of *OslRT1*, *OsPCR1*, and *OsMTP1* genes hindered Cd uptake by rice plants, which was especially evident in low-accumulating Cd rice varieties. These results provide an important theoretical reference and scientific basis for our in-depth study and understanding of the mechanism of cadmium stress tolerance in rice.

## 1. Introduction

Cadmium (Cd) is a heavy metal that is highly toxic to humans. Cd can be hidden in the human body for decades and enters the body mainly through the food chain [1,2]. After entering the human body, Cd accumulates in the body, and, even if the intake of Cd is very slight, it will also cause different degrees of harm to various organs, causing malfunction of the human body. In view of this, the World Health Organization has classified cadmium as a class I carcinogen [3,4]. Rice is one of the main food crops in the world. When planted in cadmium-contaminated fields, excessive cadmium will accumulate in large amounts in its roots, stems, leaves, and grains, which not only leads to stunted rice and low yield but also affects the quality and safety of rice, which in turn endangers human health [5,6,7].

Studies have shown that there is also a link between the regulation of Cd stress by enzyme systems. When plant organs age or suffer damage under adversity, membrane lipid peroxidation often occurs. Malondialdehyde (MDA) is the final breakdown product of membrane lipid peroxidation, the level of which can reflect the degree of plant damage from adversity [8,9,10]. After MDA is released from the sites produced on the membrane, it can react with proteins and nucleic acids, thus losing its function, and can also relax the bridge bonds between cellulose molecules or inhibit protein synthesis. MDA is also associated with reactive oxygen species (ROS), and the production of these substances increases the levels of hydrogen peroxide (H_2_O_2_) and MDA in plants [11].

The mechanism of the antioxidant system in rice is balanced with ROS production. Glutathione (GSH) is an important antioxidant substance. Plants can synthesize GSH to slow down cellular oxidative damage, which has the effect of promoting growth and improving plant tolerance to adverse environments [12]. In addition, exogenous addition of GSH not only alleviates the uptake of Cd in barley [13] and rice grainlings [14] but also decreases the accumulation of H_2_O_2_ and MDA. GSH can be divided into two main types: reduced and oxidized. Both can be interconverted through redox reactions, and this conversion process generally occurs in the corresponding organelles [15]. It has been reported that oxidized glutathione (GSSH) requires reduction to exert its effects. Cd stress promotes GSSH synthesis and increases the GSH/GSSH ratio in rice [16]. Peroxidase (POD) is a class of oxidoreductases that catalyzes many reactions and is a highly active enzyme in plants. POD is involved in reactions such as respiration, photosynthesis, and ROS metabolism. The level of POD content can also be used as an indicator of normal development in rice. Catalase (CAT) is an enzyme that catalyzes the breakdown of H_2_O_2_ into oxygen and water, thus saving the cell from H_2_O_2_ poisoning, and is one of the key enzymes of the biodefense system, present in the peroxisome of the cell [17,18]. CAT plays a key role in plants under growth and adversity stresses [19]. Superoxide dismutase (SOD) catalyzes the dismutation of superoxide anion (O_2_^−^) into H_2_O_2_ and O_2_ with antioxidant and anti-aging effects. Overexpression of SOD in transgenic plants improves plant resistance to harsh environments to varying degrees [20].

In addition to the enzyme system, high expression of some genes in different parts of rice can also enhance or hinder Cd uptake and transport. *OsNRAMP5*, identified as a key gene for Cd transport, is expressed in roots to mediate Cd and some trace elements [21]. Cd can enter the root system through ion channels mediated by other transporters. *OslRT1* was first shown to be an Fe transporter protein, and later it was found that *OslRT1* also enhances the uptake of Cd transport in the presence of Fe deficiency in the soil [22]. *OsHMA2*, expressed in stem nodes and bast, transports both Cd and Zn, and its overexpression was shown to reduce Cd content in grains [23]. It transports Cd to rice grains through Ca^2+^ channels [24]. Many other Cd transporter genes were identified, such as *OsHMA3*, *OsZIP1*, *OsPCR1* [25], etc., which act in different parts of rice, and high expression of some genes enhances Cd uptake in rice, while others act as a hindrance. Therefore, the present study, with the validation of genes at different sites based on the enzyme system, aimed to investigate the intrinsic mechanism of their co-regulation of Cd.

## 2. Results

### 2.1. Rice Variety Screening

#### 2.1.1. Screening Results of Rice Varieties in 2021

The screening results of *japonica* rice varieties are presented in Table S1. The average fluctuation range of grain Cd content of *japonica* varieties was 0.0242–0.3720 mg·kg^−1^. The high-accumulation Cd *japonica* varieties were YY4949, S4699, and ZXJ-1; the total cadmium content was 0.3720 mg·kg^−1^, 0.2431 mg·kg^−1^, and 0.2181 mg·kg^−1^. The low-accumulation *japonica* varieties were TLY619, ZN29, FJ908, and NJ5055; the total cadmium content was 0.0242 mg·kg^−1^, 0.0251 mg·kg^−1^, 0.0257 mg·kg^−1^, and 0.0286 mg·kg^−1^.

The screening results of *indica* rice varieties are presented in Table S2. The average fluctuation range of Cd content of *indica* rice varieties was 0.0351–0.3790 mg·kg^−1^. The high-accumulation Cd *japonica* varieties were JHY1586, QLY155, PY5627, and FLY1252; the total cadmium content was 0.3790 mg·kg^−1^, 0.3378 mg·kg^−1^, 0.2884 mg·kg^−1^, and 0.2864 mg·kg^−1^. The low-accumulation *japonica* varieties were G8YXSM, MXZ-2, IIY838, and LY2152; the total cadmium content was 0.0351 mg·kg^−1^, 0.0568 mg·kg^−1^, 0.0748 mg·kg^−1^, and 0.0806 mg·kg^−1^.

#### 2.1.2. Screening Results of Rice Varieties in 2022

The screening results of *japonica* rice varieties are presented in Table S3. The average fluctuation range of grain Cd content of *japonica* varieties was 0.0730–0.6541 mg·kg^−1^. The high-accumulation Cd *japonica* varieties were S4699, TLY619, and SXJ100; the total cadmium content was 0.3720 mg·kg^−1^, 0.6541 mg·kg^−1^, and 0.6369 mg·kg^−1^. The low-accumulation *japonica* varieties were YY4949, CYJ-7, FJ908, and NJ5055; the total cadmium content was 0.0730 mg·kg^−1^, 0.1603 mg·kg^−1^, 0.0920 mg·kg^−1^, and 0.0948 mg·kg^−1^.

The screening results of *indica* rice varieties are presented in Table S4. The average fluctuation range of Cd content in *indica* rice varieties was 0.0946-0.7087 mg·kg^−1^. The high-accumulation Cd *japonica* varieties were JHY1586, QLY155, and JHY8; the total cadmium content was 0.0787 mg·kg^−1^, 0.4530 mg·kg^−1^, 0.4891 mg·kg^−1^, and 0.6956 mg·kg^−1^. The low-accumulation *japonica* rice varieties include G8YXSM, MXZ-2, and TYXZ; the total cadmium content was 0.0948 mg·kg^−1^, 0.1487 mg·kg^−1^, and 0.1169 mg·kg^−1^.

**Figure 1:**
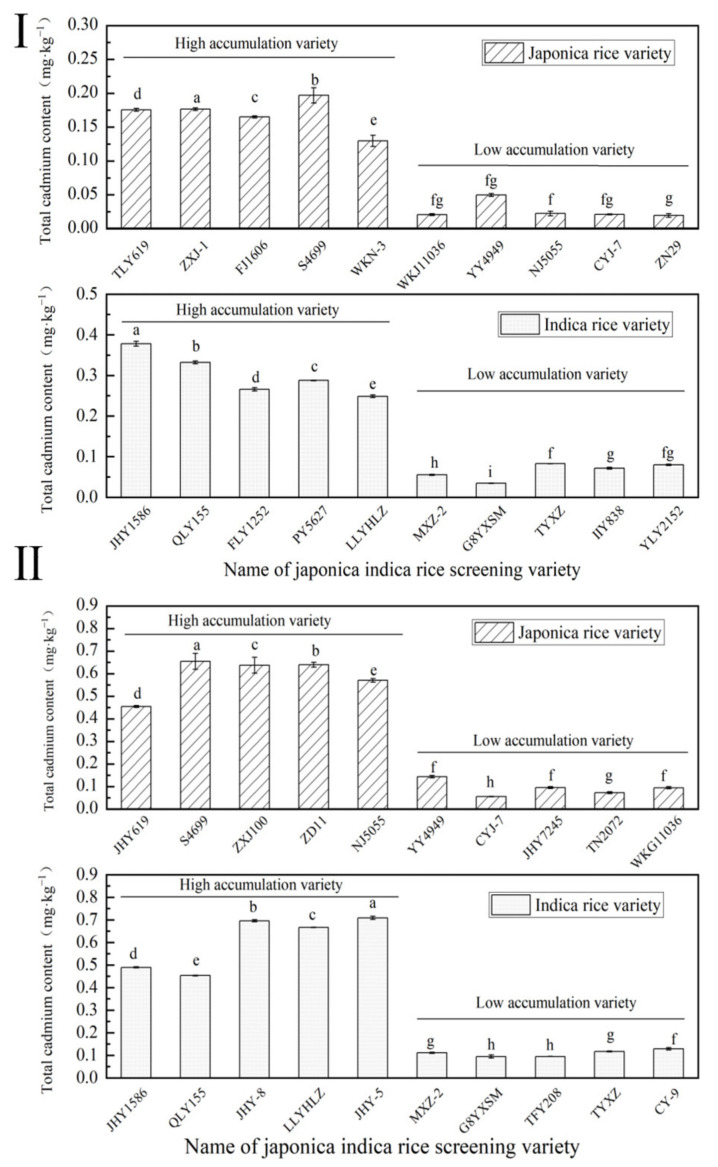
Results of rice variety screening for two years. (**Ⅰ**) 2021. (**Ⅱ**) 2022. Different letters indicate significant differences (LSD test, *p* < 0.05). The error bar represents the standard deviation of three biological replicates.

The screening results of the two years of field data of the varieties were integrated, and finally the varieties with high accumulation of Cd were screened: JHY1586, QLY155, TLY619, and S4699, and the varieties with low accumulation of Cd: MXZ-2, G8YXSM, YY4949, and CYJ-7 (Figure 1).

### 2.2. Cd Kinetic Uptake in Various Organs of Rice

**Figure 2:**
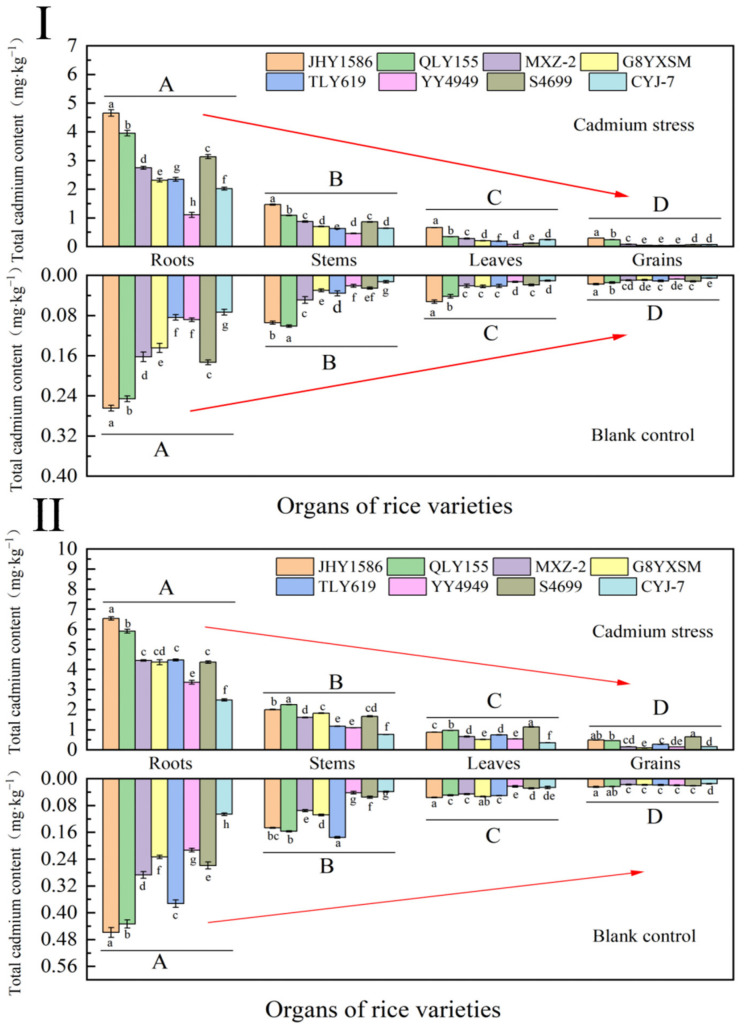
Total cadmium content in organs of rice varieties in different periods: (**Ⅰ**) heading stage and (**Ⅱ**) mature stage. Cadmium stress means cadmium stress treatment. Blank control means no cadmium stress treatment. The uppercase letters (A, B, C, and D) and lowercase letters (a, b, c, d, e, f, g, and h) indicate significant differences (LSD test, *p* < 0.01 and *p* < 0.05, respectively). JHY1586, QLY155, MXZ−2, G8YXSM, TLY619, YY4949, S4699, and CYJ−7 represent Jia he you 1586, Q liang you 155, Mei xiang zhan No.2 and Guang 8 you xiang si miao, Tian long you 619, Yong you 4949, Su 4699, and Chang you jing No.7, respectively. The red arrow shows the trend from high to low total cadmium content in roots, stems, leaves, and grains. The error bar represents the standard deviation of three biological replicates.

There were differences in total Cd content among rice varieties in Cd-stressed samples at the heading stage (Figure 2). Differences existed between different organs of the same variety, showing root (2.7830 mg·kg^−1^) > stem (0.8387 mg·kg^−1^) > leaf (0.2637 mg·kg^−1^) > grain (0.1077 mg·kg^−1^); differences existed between the same organs of different varieties, showing high accumulation > low accumulation; highly significant differences existed between different organs of different varieties. There was a difference between the same organs of different varieties, showing high accumulation > low accumulation, and a highly significant difference between different organs of different varieties, also showing root > stem > leaf > grain. The total cadmium content of all organ parts of the blank samples was lower than that of the cadmium-stressed samples. Except for the above, the rest of the overall trend was the same as that of the Cd-stressed samples.

At mature stage, Cd stress and total Cd content in blank samples were higher than those at heading stage. Other trends were the same as at heading stage. The total Cd content in all organ parts of the blank samples was significantly lower than that of the cadmium-stressed samples. The overall trends were the same as those of the Cd-stressed samples, except for the leaves and grains of YY4949, S4949, and CYJ-7, which were not significantly different. This may be due to the different Cd uptake and translocation mechanisms among different varieties.

### 2.3. Malondialdehyde (MDA) Content in Rice

**Figure 3:**
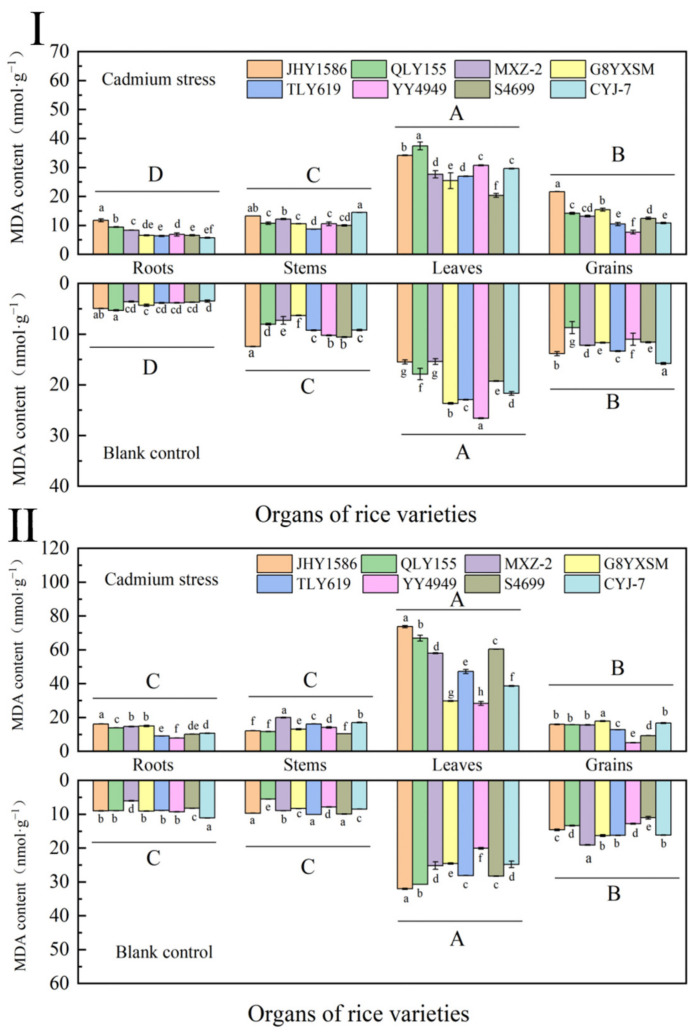
Content of malondialdehyde (MDA) in rice organs at different periods: (**Ⅰ**) heading stage and (**Ⅱ**) mature stage. Cadmium stress means cadmium stress treatment. Blank control means no cadmium stress treatment. The uppercase letters (A, B, C, and D) and lowercase letters (a, b, c, d, e, f, g, and h) indicate significant differences (LSD test, *p* < 0.01 and *p* < 0.05, respectively). JHY1586, QLY155, MXZ−2, G8YXSM, TLY619, YY4949, S4699, and CYJ−7 represent Jia he you 1586, Q liang you 155, Mei xiang zhan No.2 and Guang 8 you xiang si miao, Tian long you 619, Yong you 4949, Su 4699, and Chang you jing No.7, respectively. The error bar represents the standard deviation of three biological replicates.

The accumulation of MDA in different rice varieties in both Cd-stressed and blank samples was different (Figure 3). In the leaves of rice varieties, MDA accumulation was higher in rice varieties with low cadmium accumulation. In the Cd-stressed and blank samples, the same rice variety exhibited the accumulation of MDA in leaves (29.0351 mmol·g^−1^) > grains (13.2123 mmol·g^−1^) > stems (11.2989 mmol·g^−1^) > roots (7.6893 mmol·g^−1^). The MDA content of all organs of the Cd-stressed samples was significantly higher than that of the blank samples. This indicated that, under the stress condition of Cd, MDA content of rice organs would be increased.

The MDA content of all organs of rice was higher at the mature stage than at the heading stage. However, the trend among its varieties and organs was the same as that of the mature stage. In moderately Cd-contaminated fields, MDA accumulation in rice increased with the increase in rice reproductive process.

### 2.4. Rice Antioxidant Enzyme System

#### 2.4.1. Determination of Reduced Glutathione (GSH) in Rice at Various Periods

**Figure 4:**
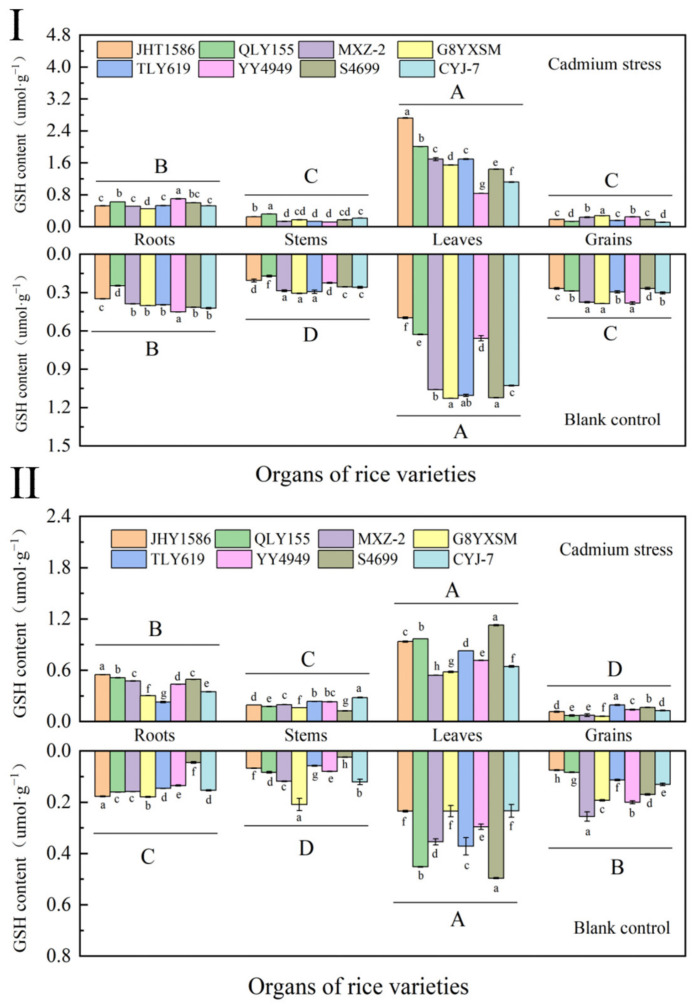
Content of glutathione (GSH) in rice organs at different periods: (**Ⅰ**) heading stage and (**Ⅱ**) mature stage. Cadmium stress means cadmium stress treatment. Blank control means no cadmium stress treatment. The uppercase letters (A, B, C, and D) and lowercase letters (a, b, c, d, e, f, g, and h) indicate significant differences (LSD test, *p* < 0.01 and *p* < 0.05, respectively). JHY1586, QLY155, MXZ−2, G8YXSM, TLY619, YY4949, S4699, and CYJ−7 represent Jia he you 1586, Q liang you 155, Mei xiang zhan No.2 and Guang 8 you xiang si miao, Tian long you 619, Yong you 4949, Su 4699, and Chang you jing No.7, respectively. The error bar represents the standard deviation of three biological replicates.

GSH was affected by Cd stress in rice leaves during the heading stage (Figure 4). In both Cd stress and blank samples, the leaves of rice varieties showed higher GSH in high-Cd-accumulation rice varieties than in low-Cd-accumulation rice varieties. The GSH content of leaves in the same variety was higher than that of all other organs, showing that leaves (1.63 μmol·g^−1^) > roots (0.5568 μmol·g^−1^) > grains (0.1899 μmol·g^−1^) > stems (0.1892 μmol·g^−1^). The GSH content in the leaves of Cd-stressed samples was higher than that of the blank samples. It proved that, in the leaves, GSH was most regulated by Cd stress.

The GSH content in all organs of rice at maturity was lower than that at mature stage. However, its content in the leaves still showed that the Cd-stressed samples were higher than the blank samples, and the GSH content in the leaves was higher than that in other organs, and this trend was the same as that in the mature stage.

#### 2.4.2. Determination of Oxidized Glutathione (GSSH) in Rice at Various Periods

**Figure 5:**
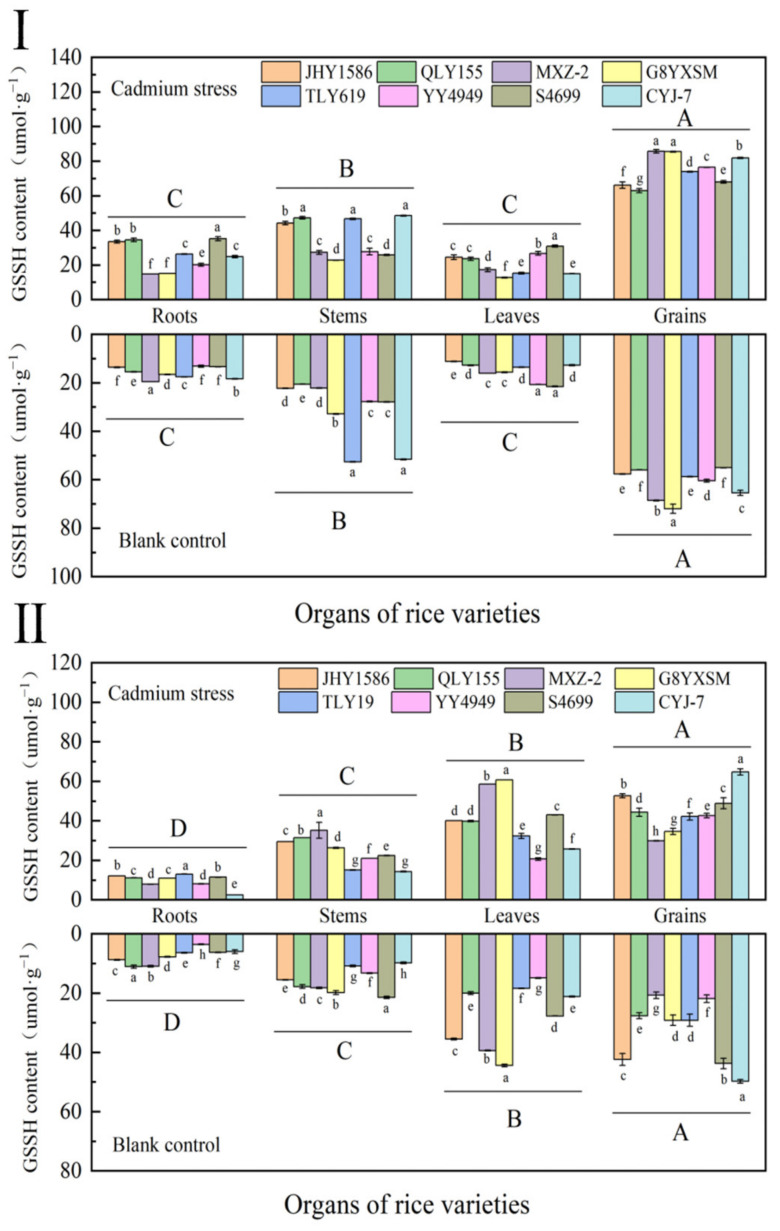
Content of oxidized glutathione (GSSH) in rice organs at different periods: (**Ⅰ**) heading stage and (**Ⅱ**) mature stage. Cadmium stress means cadmium stress treatment. Blank control means no cadmium stress treatment. The uppercase letters (A, B, C, and D) and lowercase letters (a, b, c, d, e, f, g, and h) indicate significant differences (LSD test, *p* < 0.01 and *p* < 0.05, respectively). JHY1586, QLY155, MXZ−2, G8YXSM, TLY619, YY4949, S4699, and CYJ−7 represent Jia he you 1586, Q liang you 155, Mei xiang zhan No.2 and Guang 8 you xiang si miao, Tian long you 619, Yong you 4949, Su 4699, and Chang you jing No.7, respectively. The error bar represents the standard deviation of three biological replicates.

GSSH was regulated by Cd stress in rice grains, with a negative correlation between Cd content in grains and GSSH content at the same stress level (Cd stress or blank) (Figure 5). The content of GSSH showed that grains (73.8997 μmol·g^−1^) > stems (36.2673 μmol·g^−1^) > leaves (25.5197 μmol·g^−1^) and roots (20.7137 μmol·g^−1^). Among rice varieties, it showed lower GSSH content in high-accumulation Cd rice varieties than in low-accumulation ones. The GSSH content showed higher Cd stress samples than blank samples. The degree of GSSH content in rice subjected to Cd stress also differed at different stress levels.

Compared with the mature stage, GSSH in rice organs at maturity was lower. However, it still showed a higher content in grains than in other organs, but, with the increase in the reproductive period, the GSSH content in leaves was higher than that in stems, which was as follows in the same rice variety: grains (61.2316 μmol·g^−1^) > stems (32.2454 μmol·g^−1^) > roots (15.9772 μmol·g^−1^) > leaves (15.5529 μmol·g^−1^). However, the grain GSSH content of two low-Cd-accumulation rice varieties, MXZ-2 and G8YXSM, was lower than that of high-Cd-accumulation rice varieties. This may be due to the excessive reduction of GSSH to GSH during the growth of these two rice varieties due to the influence of external growth environment, which resulted in their lower contents.

#### 2.4.3. Determination of Rice Peroxidase (POD) in Various Periods

**Figure 6:**
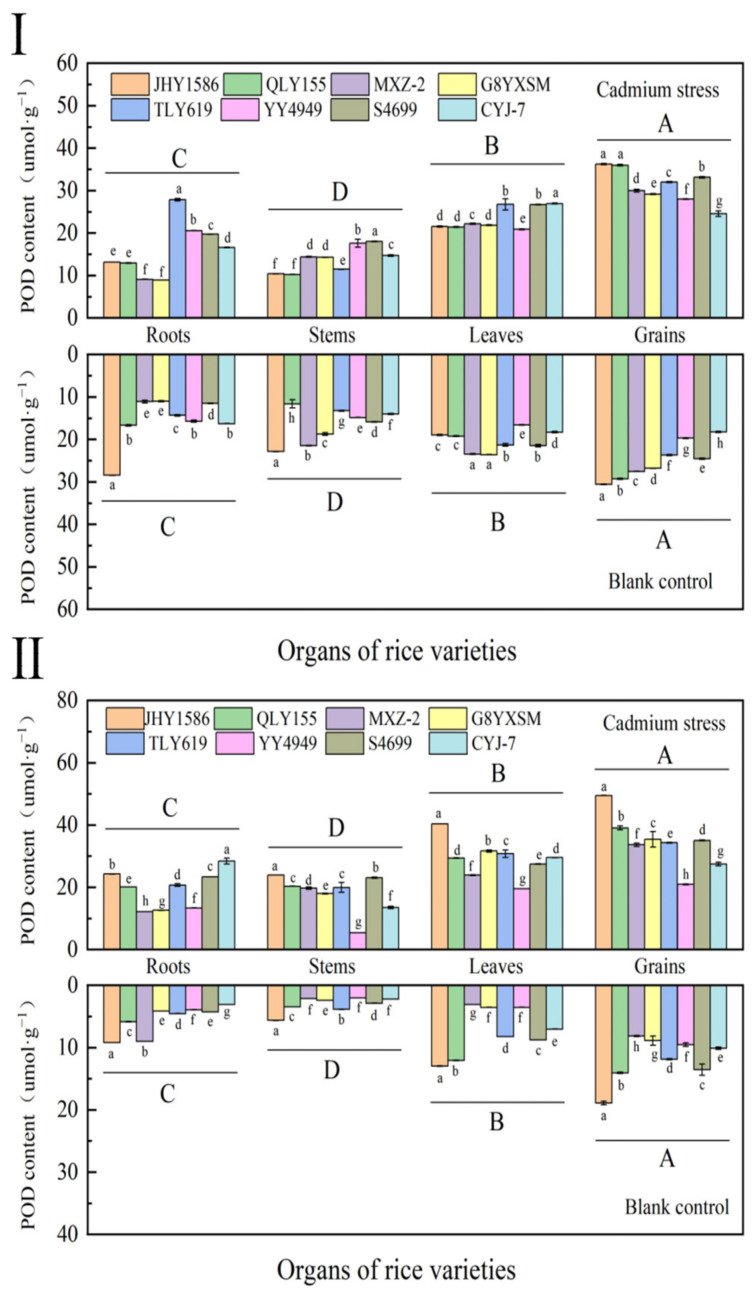
Content of peroxidase (POD) in rice organs at different periods: (**Ⅰ**) heading stage and (**Ⅱ**) mature stage. Cadmium stress means cadmium stress treatment. Blank control means no cadmium stress treatment. The uppercase letters (A, B, C, and D) and lowercase letters (a, b, c, d, e, f, g, and h) indicate significant differences (LSD test, *p* < 0.01 and *p* < 0.05, respectively). JHY1586, QLY155, MXZ−2, G8YXSM, TLY619, YY4949, S4699, and CYJ−7 represent Jia he you 1586, Q liang you 155, Mei xiang zhan No.2 and Guang 8 you xiang si miao, Tian long you 619, Yong you 4949, Su 4699, and Chang you jing NO.7, respectively. The error bar represents the standard deviation of three biological replicates.

The POD content in rice grains was higher than that in other organs at the same stress level during the heading stage (Figure 6). In the same rice variety, the content of each organ showed: grains (25.0656 μmol·g^−1^) > leaves (20.3774 μmol·g^−1^) > roots (15.6904 μmol·g^−1^) > stems (13.7001 μmol·g^−1^). Meanwhile, among different rice varieties, *indica* rice grains showed higher POD content in high-Cd-accumulation varieties than in low-Cd-accumulation varieties, while, in *japonica* rice, it was the opposite. The POD content of Cd-stressed samples was higher than that of blank samples.

In the Cd-stressed samples, the POD content of all organs of rice at mature was higher than that at tasseling stage. In contrast, in the blank samples, the POD content was lower, which may be due to the increase in fertility and the influence of external environment, thus causing a sharp decrease in POD content. The overall trend of POD content in all organs of rice at mature was the same as that of the mature stage.

#### 2.4.4. Determination of Rice Catalase (CAT) in Various Periods

**Figure 7:**
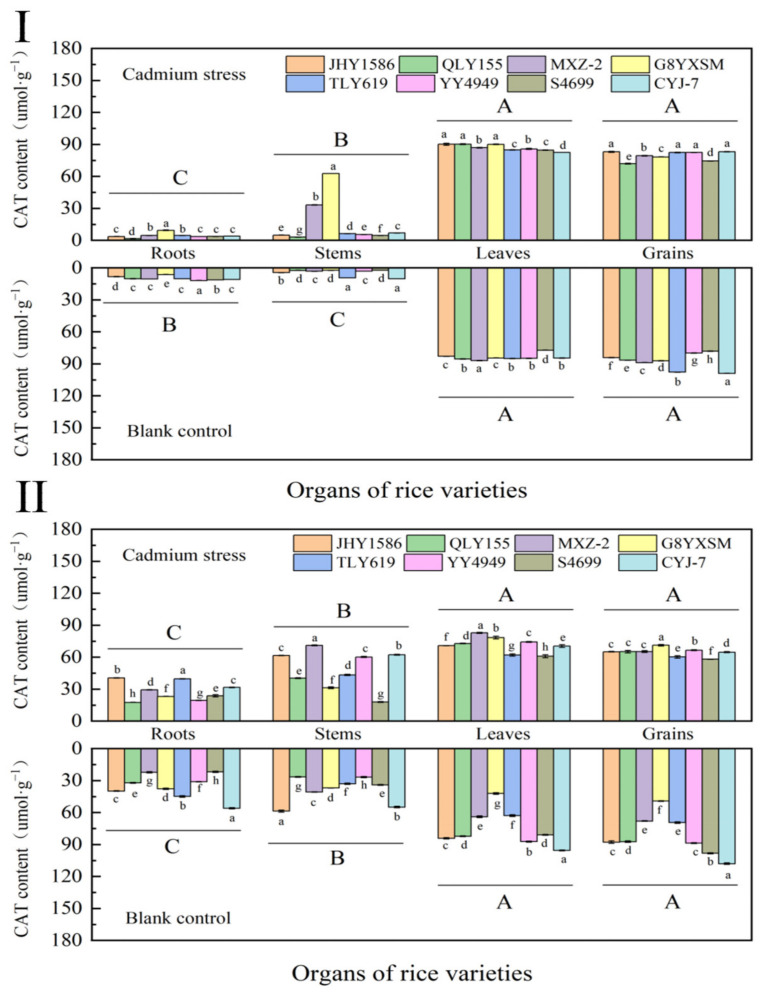
Content of catalase (CAT) in rice organs at different periods: (**Ⅰ**) heading stage and (**Ⅱ**) mature stage. Cadmium stress means cadmium stress treatment. Blank control means no cadmium stress treatment. The uppercase letters (A, B, C, and D) and lowercase letters (a, b, c, d, e, f, g, and h) indicate significant differences (LSD test, *p* < 0.01 and *p* < 0.05, respectively). JHY1586, QLY155, MXZ−2, G8YXSM, TLY619, YY4949, S4699, and CYJ−7 represent Jia he you 1586, Q liang you 155, Mei xiang zhan No.2 and Guang 8 you xiang si miao, Tian long you 619, Yong you 4949, Su 4699, and Chang you jing NO.7, respectively. The error bar represents the standard deviation of three biological replicates.

The CAT contents in leaves (84.0357 μmol·g^−1^) and grains (87.7516 μmol·g^−1^) of the same rice variety were higher than those in roots (9.9702 μmol·g^−1^) and stems (4.7183 μmol·g^−1^) during the heading stage, while there were no differences in CAT contents in leaves and grains (Figure 7). The content of CAT in each organ of different rice varieties was not regulated by Cd stress, and there was no significant difference in CAT content between Cd-stressed and blank samples.

At mature stage, the CAT content of rice leaves and grains was reduced, while the CAT content in roots and stems was increased, while the CAT content in the roots and stems increased. It was also demonstrated once again that CAT content in rice was not significantly correlated with total Cd and rice content by maturity.

#### 2.4.5. Results of Superoxide Dismutase (SOD) Measurements in Rice at Various Periods

**Figure 8:**
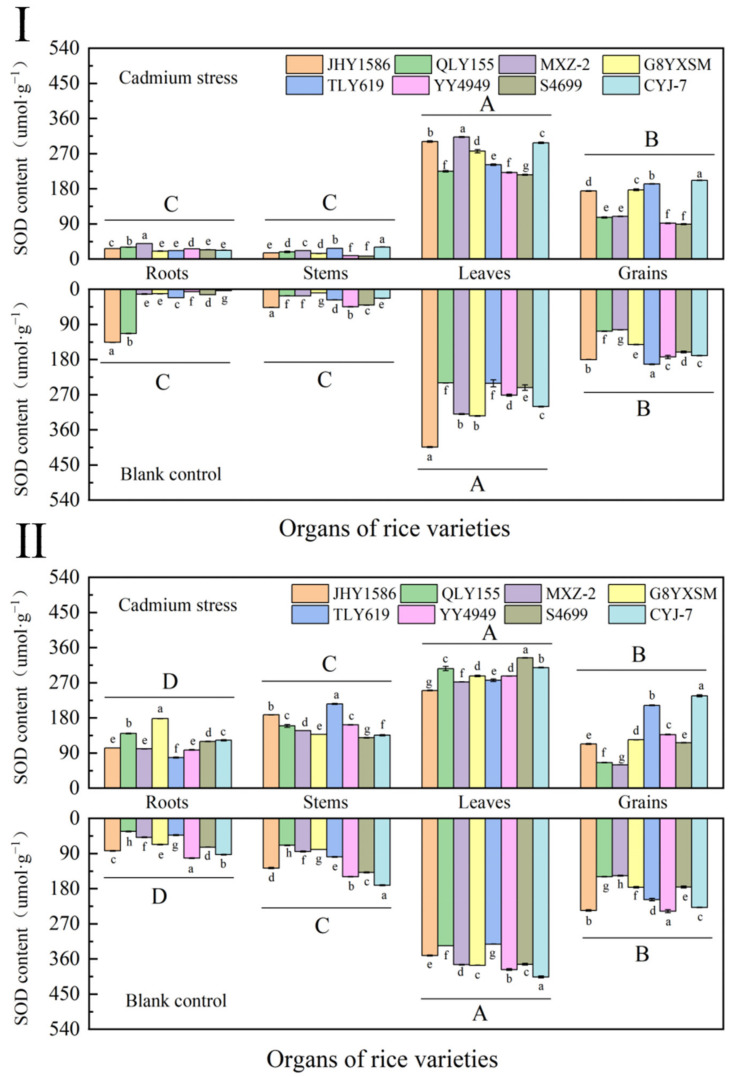
Content of superoxide dismutase (SOD) in rice organs at different periods. (**Ⅰ**) heading stage and (**Ⅱ**) mature stage. Cadmium stress means cadmium stress treatment. Blank control means no cadmium stress treatment. The uppercase letters (A, B, C, and D) and lowercase letters (a, b, c, d, e, f, g, and h) indicate significant differences (LSD test, *p* < 0.01 and *p* < 0.05, respectively). JHY1586, QLY155, MXZ−2, G8YXSM, TLY619, YY4949, S4699, and CYJ−7 represent Jia he you 1586, Q liang you 155, Mei xiang zhan No.2 and Guang 8 you xiang si miao, Tian long you 619, Yong you 4949, Su 4699, and Chang you jing NO.7, respectively. The error bar represents the standard deviation of three biological replicates.

The accumulation of SOD in rice leaves was more than in other organs (Figure 8). Among different rice varieties, it showed that the SOD content in the leaves of high-Cd-accumulation rice varieties was lower than that of low-Cd-accumulation rice varieties; among the same rice varieties, the content of SOD showed that leaves (301.0227 μmol·g^−1^) > grains (151.8823 μmol·g^−1^) > roots (37.7962 μmol·g^−1^) > stems (28.3889 μmol·g^−1^. The SOD content of Cd-stressed samples was lower than that of the blank samples. The content of SOD in rice leaves was more significantly regulated by Cd stress.

At maturity, the content of SOD in all organs of rice increased significantly. The SOD content in leaves of rice variety QLY155 was decreased. The rest showed the same trend in different rice varieties as in the mature stage.

### 2.5. Analysis of rice gene expression by RT-qPCR

**Figure 9:**
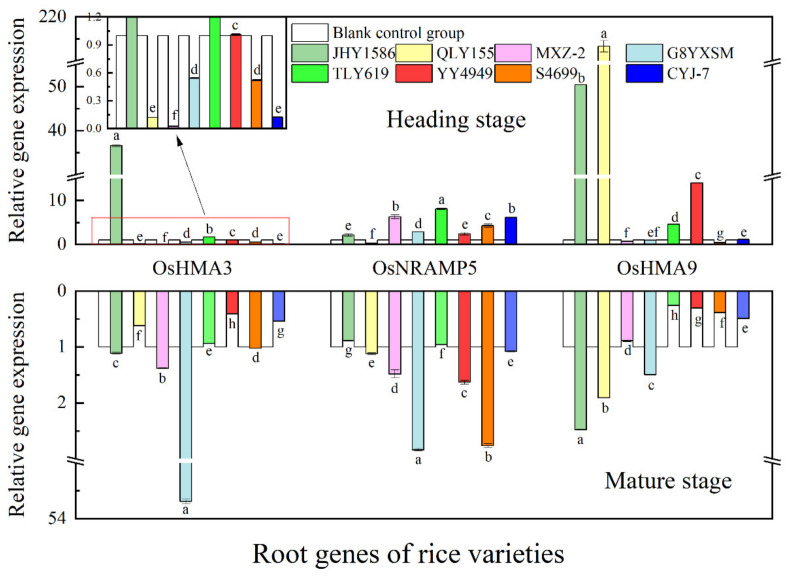
Relative gene expression at heading and mature stages of rice roots. Different letters indicate significant differences (LSD test, *p* < 0.05).

**Figure 10:**
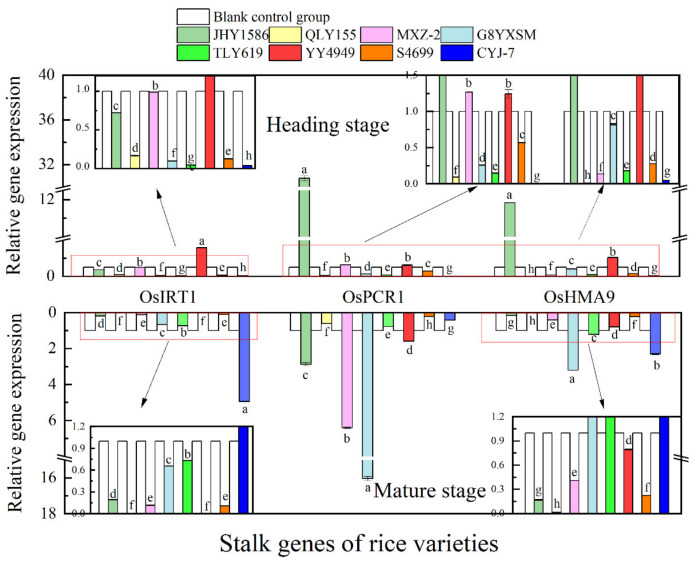
Relative gene expression in rice stem heading stage and mature stage. Different letters indicate significant differences (LSD test, *p* < 0.05). The error bar represents the standard deviation of three biological replicates.

**Figure 11:**
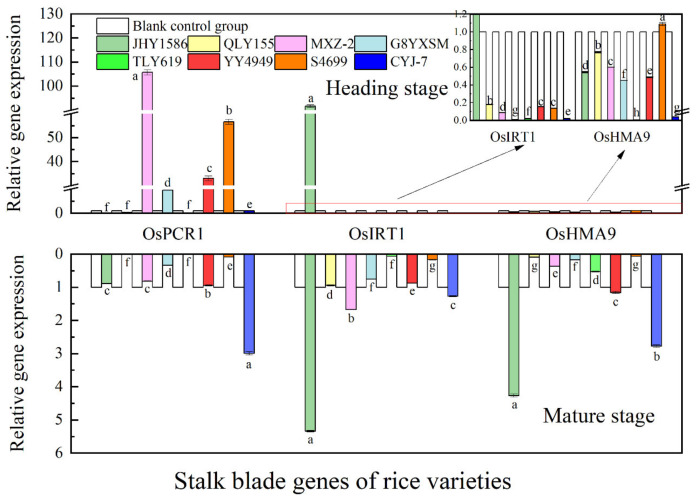
Relative gene expression in rice leaves at heading and mature stages. Different letters indicate significant differences (LSD test, *p* < 0.05). The error bar represents the standard deviation of three biological replicates.

**Figure 12:**
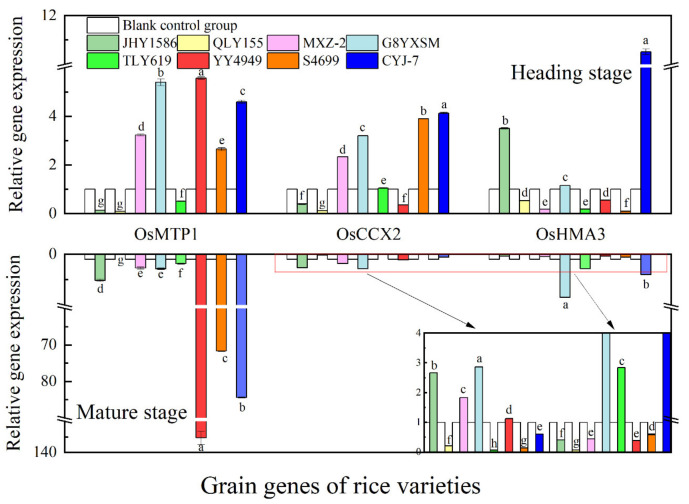
Relative gene expression in rice grain at heading and mature stages. Different letters indicate significant differences (LSD test, *p* < 0.05). The error bar represents the standard deviation of three biological replicates.

Based on the findings of previous studies, we selected 12 genes related to Cd ab-sorption and transport expressed in various organs of rice. From these 12 genes (Table S5), reference genes with high expression in rice organs were screened, and their relative gene expression levels were measured. The genes that were highly expressed in each organ part (root: *OsHMA3*, *OsNRAMP5*, and *OsHMA9*; stem: *OslRT1*, *OsPCR1*, *OsCCX2*; leaf: *OsNRAMP5*, *OsPCR1*, *OsHMA9*; grain: *OsHMA3*, *OsMTP1*, *OsCCX2*) were screened. The relative gene expression results for each organ locus of the eight rice varieties were as follows:

Regarding gene expression in root (Figure 9), OsHMA3 gene at mature stage had the highest expression in JHY1586 and OsHMA9 gene in JHY1586 and QLY155; OsHMA3 gene at mature stage had the highest expression in G8YXSM and OsNRAMP5 in G8YXSM and S4699 and OsHMA9 gene in JHY1586 and QLY155. Regarding gene expression in stem (Figure 10), OsPCR1 and OsHMA9 genes at mature stage had the highest expression in JHY1586 and OsIRT1 gene in YY4949; OsIRT1 gene at mature stage had the highest expression in CYJ-7 and OsPCR1 gene in G8YXSM and MXZ-2 and OsHMA9 gene in G8YXSM and CYJ-7. The expression of OsHMA9 gene was the highest in both G8YXSM and CYJ-7. Regarding gene expression in leaf (Figure 11), OsPCR1 gene had the highest expression in MXZ-2, YY4949, and S4699 at mature stage and OsIRT1 gene in JHY1586; OsPCR1 gene had the highest ex-pression in CYJ-7 and OsIRT1 in JHY1586 and OsHMA9 gene in JHY1586 and CYJ-7 at mature stage. Regarding gene expression in grain (Figure 12), OsMTP1 gene had the highest expression in the rice varieties at mature stage in G8YXSM, YY4949, and CYJ-7 and OsCCX2 gene in four rice varieties with low accumulation and OsHMA3 gene in JHY1586 and CYJ-7; OsMTP1 gene had the highest expression at mature stage in YY4949, S4699, and CYJ-7 and OsCCX2 in G8YXSM and OsHMA9 gene in JHY1586 and CYJ-7. OsMTP1 gene had the highest expression in YY4949, S4699, and CYJ-7; both OsCCX2 and OsHMA3 genes were the highest expressed in G8YXSM and CYJ-7.

The relative gene expression levels of blank samples of *OsHMA3*, *OsCCX2*, *OsNRAMP5*, and *OsHMA9* genes were all higher than those of Cd-stressed samples, thus indicating that the presence of these four genes in different organs of rice facilitates the uptake and translocation of Cd in the plants. It was due to the high expression of these genes that led to the increase in Cd content in various organs of the plant. Thus, these four genes play an important role in the uptake and translocation of Cd in rice varieties with high Cd accumulation. In contrast, the presence of the remaining three genes, *OslRT1*, *OsPCR1*, and *OsMTP1*, had a hindering effect on the uptake and translocation of Cd in the plants. The high expression of these three genes leads to a decrease in Cd content in rice varieties. Therefore, the relative gene expression of these three genes would be relatively high in low-accumulation Cd rice varieties.

### 2.6. Correlation Analysis

#### 2.6.1. Correlation Analysis of Total Cd and Enzymatic Activities in Various Organs of Rice at Different Periods

**Figure 13:**
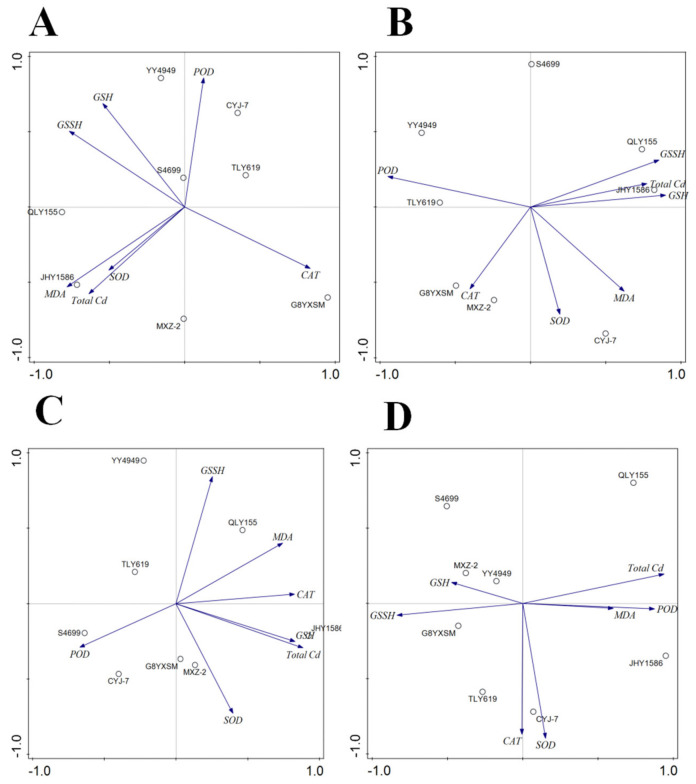
Correlation analysis between total Cd and enzyme contents of different organs of rice varieties at the heading stage ((**A**): root; (**B**): stalk; (**C**): leaf; (**D**): grain).

The correlation analysis of rice heading stage (Table 1 and Figure 13) show that, in the roots of rice, there was a highly significant correlation between total Cd and MDA content, a positive correlation between GSH and CAT content, and a negative correlation between GSSH, GSH, and CAT content. In the stalks of rice, there was a highly positive correlation between total Cd content and GSH and a positive correlation with GSSH. In the leaves of rice, there was also a highly positive correlation between total Cd and GSSH and a positive correlation with CAT. In the grains of rice, there was a positive correlation between total Cd and GSH and a negative correlation with POD.

**Figure 14:**
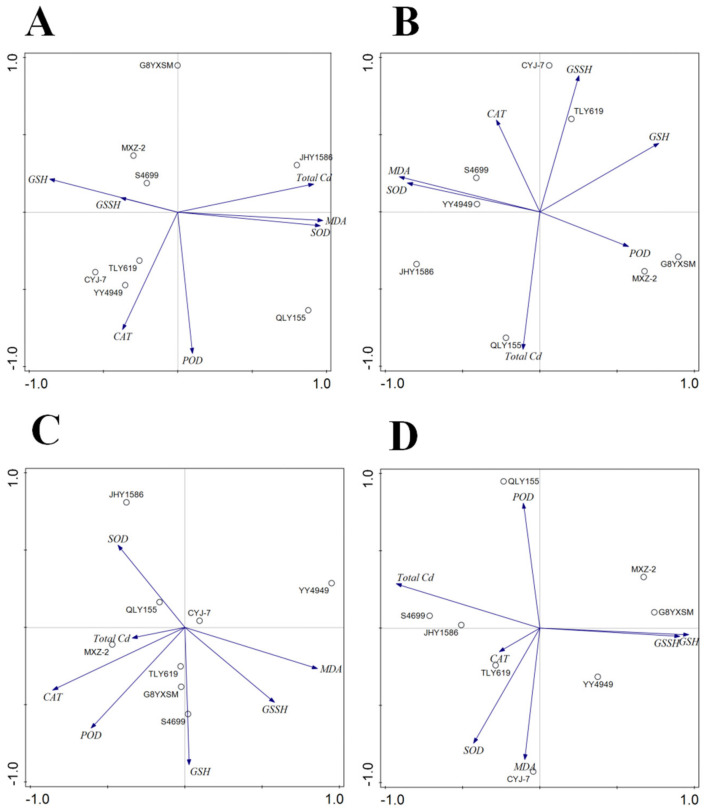
Correlation analysis between total Cd and enzyme contents of different organs of rice varieties at the mature stage: (**A**) root; (**B**) stalk; (**C**) leaf; (**D**) grain.

The correlation analysis of rice mature stage (Table 2 and Figure 14) shows that, in the roots of rice, there was a highly positive correlation between total Cd and MDA and SOD content and a negative correlation with GSH. In the stalks of rice, there was a negative correlation between total Cd and GSSH. In the leaves of rice, there was no correlation between total Cd and other indicators. In the grains of rice, there was a highly negative correlation between total Cd and GSSH and GSH.

#### 2.6.2. Correlation Analysis between Total Cd and Enzyme Activities of Rice Varieties under Different Factors

The results of the correlation analysis showed that (Table 3) there was no difference between total cadmium and enzyme activity in rice under different periods and three factors (periods, varieties, and organ) of co-cropping patterns. In contrast, differences existed between total Cd and enzyme activities in rice under both variety, organ, and two factors (period and variety) of inter-cropping patterns. In addition, there were differences between total Cd and other enzyme activities except for POD activity under the period and organ inter-cropping patterns. No differences existed between total Cd and all other enzyme activities except POD activity under variety and organ inter-cropping patterns.

## 3. Discussion

### 3.1. Low-Cd Rice Variety Screening

There were differences in the strength of Cd uptake capacity of different rice varieties. High-accumulation Cd varieties have higher Cd uptake and enrichment capacity compared to other varieties, which leads to excessive Cd content in rice grains after maturity. Therefore, the screening of low-accumulation rice varieties has received extensive attention. Currently, there are two screening methods for low-Cd varieties: conventional screening and molecular-marker-assisted screening. Conventional screening is one of the most common methods. Hui et al. [26] used the conventional screening method through potting experiments to screen 43 rice varieties for low cadmium and contamination safety and found that 30 of them were cadmium contamination safety varieties. Li et al. [27] conducted a comprehensive screening of 32 rice varieties for low Cd, low Pb, micronutrient content, and yield through pot experiments and finally screened three rice varieties to meet the screening conditions. Xu et al. [28] screened low-Cd rice varieties by field experiments while assessing the environmental threshold of Cd in farmland in a specific region and finally deduced that the soil environmental Cd threshold in the region ranged from 0.89 to 24.39 mg-kg^−1^ and screened five low-Cd rice varieties. Sun et al. [29] used a molecular-marker-assisted approach to identify rice varieties through field trials and six rice varieties were identified as low-Cd varieties.

Different scholars have used different methods to screen low-Cd rice varieties. In this study, we also used the conventional screening method; the total Cd content of 96 rice varieties was compared, and four varieties with high Cd accumulation (S4699, TLY619, JHY1586, QLY155) and four varieties with low Cd accumulation (YY4949, CYJ-7, G8YXSM, MXZ-2) were identified by measuring the total cadmium content in brown rice.

### 3.2. Response of Enzyme Contents to Cadmium Absorption and Transport

The physiological mechanisms of Cd uptake transport and enrichment in rice have been well studied. It has been shown [30] that cadmium enters rice roots through ion channels, passes through xylem and phloem, and then accumulates in stems, leaves, and grains. Therefore, to investigate the intrinsic mechanism of Cd uptake differences in rice, a comprehensive analysis of enzyme contents in various organs of rice is required.

Under cadmium stress, there are significant differences in the regulation of cadmium by some enzymes of the oxidative and antioxidant series, with a significant increase in reactive oxygen species in vivo, causing varying degrees of damage to cell membranes [31]. This leads to an increase in MDA activity and disruption of cellular metabolism [32]. Chen et al. [33] analyzed the effect of cadmium stress on the enzyme activity content of MDA, SOD, and CAT in rice grainlings through hydroponic experiments and showed that cadmium stress in rice grainlings leads to an increase in MDA and POD content and a decrease in SOD and CAT content. Shao et al. [34] investigated the trends of antioxidant enzyme systems in rice under different Cd stress concentrations through hydroponic experiments. The results showed that the contents of SOD, CAT, and POD decreased with increasing Cd stress concentrations, while MDA was the opposite. Xu et al. [35] aimed to reduce Cd in rice leaves by assessing the protective effect of nitric oxide against Cd stress in rice leaves. The results showed that MDA content, SOD, CAT, and POD contents were significantly increased under CdCl2 treatment conditions. Adam Sheka Kanu et al. [36] used pot experiments to investigate the changes in enzymatic antioxidant enzyme contents in rice under Cd stress and showed that photosynthetic pigments were significantly reduced, H_2_O_2_ and MDA contents were increased, and SOD, POD, and CAT contents were decreased. Javaria Afzal et al. [37] used hydroponic experiments to study the effects of cadmium on rice growth and antioxidant enzyme mechanisms and showed that cadmium stress induced oxidative stress markers, decreased MDA and H_2_O_2_ contents, and also increased the contents of enzymatic antioxidants, SOD, CAT, POD, and APX enzymes.

In addition, some enzymes of the GSH family are equally relevant for cadmium. It was shown [35] that GSH content in rice leaves was significantly reduced under Cd stress. Michiel Huybrechts et al. [38] placed rice after one day of Cd exposure, resulting in flocculation in oxidative homeostasis in roots and leaves and an increase in genes related to GSH metabolism. Related studies have shown [39,40] that exogenous addition of GSH in hydroponic nutrient solution or in soil also increases Cd uptake by rice stems, leaves, and grains, and the results may vary among rice varieties and Cd concentrations.

In the present study, based on previous studies, we analyzed the effects of Cd stress on the contents of six enzymes in each organ of different rice varieties at the heading and mature stages. Most of the previous studies were conducted by hydroponics and potting, while the present study used field experiments to investigate the results obtained under Cd stress. Among the oxidative and antioxidant series of enzymes, under cadmium stress, MDA content and POD enzyme activity increased, SOD activity decreased, and CAT activity did not differ significantly from the results of previous studies, probably because both rice varieties and Cd stress concentrations were different. Among the glutathione series, under cadmium stress, both GSH and GSSH contents were increased, and their contents were significantly higher under cadmium stress than blank stress.

### 3.3. Changes in Expression of Cd Transporter Genes in Rice in Response to Cd Stress

The mechanism of Cd stress tolerance in rice is a complex biological process that involves not only the physiological level but also its gene regulation closely. Several genes related to Cd accumulation have been identified. These include genes of the natural-resistance-associated macrophage protein (*NRAMP*) family, genes of the P-type heavy metal ATPase (*HMA*) family, and other genes involved in Cd uptake and transport. Among them, the *OsNRAMP5* gene of the *NRAMP* family is an important gene involved in Cd uptake by rice roots [41]. Its mutation restricts Cd ion uptake by roots, thus reducing Cd accumulation in rice, and is a key gene in the selection of low-Cd rice varieties [42,43]. In addition, it mediates the translocation of Mn and Fe ions from the roots to above ground and their accumulation in the grains, maintaining the dynamic balance of Mn and Fe ions in rice [21,44]. *OsNRAMP1* and 2 in the *NRAMP* family are also involved in the translocation and accumulation of Cd [45,46]. In contrast, *OsNRAMP3* and 4 can transport other metal ions, such as Mn, Fe, and Al, but not Cd [47,48].

*OsHMA2* and *OsHMA3* of the *HMA* family were identified as key genes involved in Cd translocation [49]. *OsHMA3* further reduces Cd translocation to above ground by translocating Cd absorbed by the root system to the vesicles for storage, thereby reducing Cd content above ground as well as Cd accumulation in the grains of rice [50,51]. *OsHMA2* mediates the translocation of Cd and Zn from the root to above ground and xylem to phloem [23]. In addition, there are other families involved in Cd uptake and translocation. *OsIRT1* and *OsIRT2* of the *OsZIP* family also mediate Cd uptake and translocation, and overexpression increases sensitivity to excess Cd [52]. The presence of *OsMTP1*, a member of the rice metal tolerance protein gene family (*MTP*), was shown to enhance plant tolerance to Cd [53]. *OsCCX2* is a calcium/cation reverse transporter gene, and its presence alters Cd partitioning across organs but reduces Cd content in the grains [24].

The results showed that the trend of Cd regulation by these genes in rice varieties coincided with the previously studied genes, but the present study further analyzed some differences in high- and low-accumulating Cd rice varieties and among organs of rice varieties. High expression of *OsHMA3*, *OsCCX2*, *OsNRAMP5*, and *OsHMA9* genes might promote the uptake and translocation, whereas high expression of *OslRT1*, *OsPCR1*, and *OsMTP1* genes might hinder the uptake of Cd by rice plants.

The results of relative gene expression further verified the differences in physiological indicators, and high contents of MAD, reduced GSH, GSSH, POD, and high expression of *OsHMA3*, *OsCCX2*, *OsNRAMP5*, and *OsHMA9* genes all might promote Cd uptake and transport in rice. High activity of SOD and high expression of *OslRT1*, *OsPCR1*, and *OsMTP1* genes might hinder the uptake of Cd in rice.

How to reduce the cadmium content in rice cannot be generalized. Firstly, we should study the mechanisms of cadmium absorption, transport, and enrichment in different genotypes of rice varieties and, on this basis, accelerate the selection and breeding of new varieties with low cadmium in order to effectively solve the problem of excessive cadmium content in rice and provide a theoretical basis for safe rice production.

## 4. Materials and Methods

### 4.1. Experimental Material Cultivation and Screening

Rice varieties were provided by the Rice Research Institute of Anhui Academy of Agricultural Sciences in China. Preliminary rice variety cultivation and screening were conducted in a mildly Cd-contaminated farmland (total Cd: 0.48 mg·kg^−1^) and a moderately Cd-contaminated farmland (total Cd: 1.2 mg·kg^−1^) in Tongling City, Anhui Province, China. In mid-May 2021, the rice grains to be planted by variety were soaked in breeding bags until they showed white, and then the grains were sown onto grainling trays and put into a field free of Cd contamination to cultivate grainlings. On 16 and 17 June 2021, the transplanting of rice grainlings was carried out, and *Indica* and *japonica* rice was planted into lightly Cd-contaminated fields. There were three plots of each variety (one replicate per plot, total three replicates), each plot having an area of 6 m^2^ (6 m long by 1 m wide). On 7 October 2021, sampling of varieties of grains was carried out during the heading and mature stages. The same method was used for rice planting in 2022.

### 4.2. Determination of Total Cadmium Content and Antioxidant Enzyme Contents

The root, stem, leaf, and grain parts of rice were collected during the mature stage and mature stage, respectively. Samples for total Cd determination were cleaned after retrieval, put into an oven at 105 °C for 30 min to kill plant tissues, baked at 80 °C until constant weight and removed to grind into powder (passed through 10 mesh sieve), and stored in self-sealing bags for measurement. Samples for physiological determination need to be processed in the field, so carry a 10L bottle of liquid nitrogen tank when taking samples, remove the rice from the field and rinse it with water, divide it into different parts, and put it into the liquid nitrogen tank for temporary storage, and put it into −80 °C refrigerator for storage after retrieval. The total cadmium was determined by wet digestion method. Further, 0.5 g plant organ powder was weighed, and 9 mL nitric acid and 1 mL perchloric acid were added and soaked overnight in a sterilizing tube. The next day, use constant temperature sterilization furnace 180 °C for high-temperature sterilization for 3 h. When the solution in the tube becomes colorless and transparent, take it out and use 1% dilute nitric acid to set volume to 50 mL. The liquid with constant volume was determined by atomic absorption spectrophotometer (OD value: 228.8 nm). The enzyme activity was determined using six kits (MDA, GSH, GSSH, CAT, POD, and SOD content test kits) from Suzhou Kemin Biotechnology Co., Ltd, China. (www.cominbio.com, accessed on 15 March 2023).

The determination principle was as follows: weigh 0.1 g of fresh rice organs (three biological replicates), grind them with 1 mL of extract, the ground extract is placed into 2mL EP tube with lid, and store them on ice after finishing. After pre-treatment with the kit, the content was determined with the enzyme−label instrument. The absorbance values of GSH, GSSH, POD, CAT, and SOD extract at the wavelengths of 412 nm, 412 nm, 470 nm, 405 nm, and 560 nm were determined, respectively. The absorbance value of MDA extract is obtained by subtracting the absorbance value of 600 nm from the absorbance value of 532 nm. The specific determination method is as follows (Table 4). 

### 4.3. Gene Expression Analysis

The 12 pairs of primers were designed by Nanjing DynaScience Biotechnology Co., Ltd. (www.tsingke.net, accessed on 15 March 2023). Total RNA was extracted by Trizol method; total RNA (1 μg) was reverse-transcribed with reverse transcriptase Beijing Adera Biological Co., Ltd. (www.aidlab.cn, accessed on 15 March 2023) to obtain cDNA. BeastarTM Real-time PCR Master Mix SYBR Green (DBI Bioscience, https://www.xinghanbio.com, accessed on 15 March 2023) was used as PCR Mix, and the Mastercycler EP Realplex RT-PCR system (Eppendorf, https://www.eppendorf.com, accessed on 15 March 2023) to detect expressed gene levels. To determine the differences in relative changes among samples in each experiment, the Ct value of *β-actin* was used for data normalization, and the 2^−ΔΔCt^ method [54] was used to calculate the relative changes.

### 4.4. Statistical Analysis

The experimental data were processed, calculated, and counted using WPS office, SPSS 25.0 for ANOVA, correlation analysis, and significance of differences test, and the graphs were generated using origin 2022.

All error lines in the graphs of this paper are the standard deviations of three biological replicates of the data; capital letters (A, B, C, D) indicate significant differences between different organs; lowercase letters in the graphs (a, b, c, d) indicate significant differences between the same organs of different varieties.

## 5. Conclusions

In this study, we screened *japonica* rice high accumulation: S4699, TLY619, low accumulation: YY4949, CYJ-7; *indica* rice high accumulation: JHY1586, QLY155, low accumulation: G8YXSM, MXZ-2. The total Cd content of different rice varieties showed that *indica* rice > *japonica* rice. The total Cd content of each organ of rice showed as follows: root > stem > leaf > grain. Further study revealed that MAD, reduced GSH, GSSH, and POD were more active in high-accumulating rice varieties, and it is known that the high activity of these four enzymes enhances the uptake of Cd in rice. SOD, on the contrary, was more active in low-accumulation rice varieties, and its high activity diminished Cd uptake in rice. In contrast, there was no correlation between CAT activity and total Cd content in rice. The contents of rice MDA, POD, and SOD increased with increasing fertility. The opposite was true for reduced GSH and GSSH. CAT content in rice leaves and grains decreased with the increase in growth period, while CAT content in roots and stems increased with the increase in growth period. The high expression of OsHMA3, OsCCX2, OsNRAMP5, and OsHMA9 genes might promote the absorption and transport of cadmium in rice. High expression of OslRT1, OsPCR1, and OsMTP1 genes might hinder Cd absorption in rice. However, other roles played by MDA and antioxidant enzyme contents in rice Cd stress and specific ways of gene regulation of Cd uptake and translocation have not been fully explored in the current study and need to be followed up with further studies. 

## Figures and Tables

**Table 1:** Correlation analysis of total Cd and enzyme activity of each organ at the mature stage of rice.

Time/ Organ	Index	Total Cadmium	MDA	GSH	GSSH	POD	CAT	SOD
MS Root	Cd	1.0000	0.83 **	−0.2100	0.2400	−0.4400	−0.3300	0.2700
	MDA		1.0000	0.0000	0.4300	−0.5100	−0.3600	0.4700
	GSH			1.0000	0.74 *	0.3000	−0.68 *	0.1100
	GSSH				1.0000	0.3100	−0.72 *	−0.0500
	POD					1.0000	−0.3400	−0.4600
	CAT						1.0000	−0.3800
	SOD							1.0000
MS Stalk	Cd	1.0000	0.2700	0.89 **	0.75 *	−0.96 **	−0.6300	−0.4900
	MDA		1.0000	0.3900	0.5000	−0.98 **	−0.3700	0.4900
	GSH			1.0000	0.9100 **	−0.98 **	−0.82 *	−0.0800
	GSSH				1.0000	−0.96 **	−0.96 **	−0.1100
	POD					1.0000	0.1800	−0.6500
	CAT						1.0000	−0.1500
	SOD							1.0000
MS Leaf	Cd	1.0000	0.5900	0.90 **	0.0100	−0.3100	0.67 *	0.5500
	MDA		1.0000	0.4200	0.0300	−0.4900	0.6200	0.1100
	GSH			1.0000	0.0600	−0.2400	0.69 *	0.3200
	GSSH				1.0000	−0.0900	−0.3200	−0.5800
	POD					1.0000	−0.6200	−0.1800
	CAT						1.0000	0.3500
	SOD							1.0000
MS grain	Cd	1.0000	0.3300	0.68 *	0.5300	−0.74 *	−0.1800	−0.0900
	MDA		1.0000	0.3600	0.3900	−0.6600	−0.0500	0.3600
	GSH			1.0000	0.73 *	−0.90 **	−0.2700	0.1100
	GSSH				1.0000	−0.71 *	−0.6300	0.0400
	POD					1.0000	0.1000	−0.2200
	CAT						1.0000	−0.0600
	SOD							1.0000

*: *p* < 0.05; **: *p* < 0.01. HD: heading stage.

**Table 2:** Correlation analysis of total Cd and enzyme activity of each organ at mature stage in rice.

Time/ Organ	Index	Total Cadmium	MDA	GSH	GSSH	POD	CAT	SOD
MS Root	Cd	1.0000	0.83 **	−0.76 *	−0.3200	−0.2000	−0.3000	0.86 **
	MDA		1.0000	−0.85 **	−0.3800	0.2100	−0.4000	0.90 **
	GSH			1.0000	0.0100	−0.2600	0.1400	−0.80 **
	GSSH				1.0000	−0.0700	0.0200	−0.3800
	POD					1.0000	0.4600	0.1800
	CAT						1.0000	−0.2800
	SOD							1.0000
MS Stalk	Cd	1.0000	−0.0800	−0.4200	−0.76 *	0.0900	−0.3300	−0.1400
	MDA		1.0000	−0.4700	−0.0600	−0.3700	0.3700	0.94 **
	GSH			1.0000	0.5200	0.5900	0.0700	−0.4000
	GSSH				1.0000	−0.3100	0.3000	−0.1600
	POD					1.0000	−0.1100	−0.2500
	CAT						1.0000	0.2700
	SOD							1.0000
MS Leaf	Cd	1.0000	−0.5200	−0.1700	0.1700	0.1300	0.1900	−0.2100
	MDA		1.0000	0.3900	0.4000	−0.2800	−0.6200	−0.3500
	GSH			1.0000	0.2600	0.5300	0.3600	−0.2900
	GSSH				1.0000	0.0400	−0.4300	−0.5100
	POD					1.0000	0.69 *	0.1200
	CAT						1.0000	0.0300
	SOD							1.0000
MS grain	Cd	1.0000	−0.1700	−0.88 **	−0.82 **	0.3400	0.1700	0.2300
	MDA		1.0000	−0.1600	0.1000	−0.4300	0.2500	0.6100
	GSH			1.0000	0.82 **	−0.2100	−0.3400	−0.3100
	GSSH				1.0000	0.0300	−0.0700	−0.3500
	POD					1.0000	−0.1500	−0.4700
	CAT						1.0000	−0.1400
	SOD							1.0000

*: *p* < 0.05; **: *p* < 0.01. MS: mature stage.

**Table 3:** Correlation analysis between total Cd and enzyme activity of rice varieties under different factors.

Factor	DF	Total Cadmium	MDA	GSH	GSSH	POD	CAT	SOD
Stage (S)	1	NS	NS	NS	NS	NS	NS	NS
Cultivar (C)	7	8.8362 ***	7.7332 ***	6.5727 ***	4.0454 ***	5.1296 ***	6.0583 ***	6.3067 ***
Organ (T)	3	60.2580 ***	61.4730 **	60.2580 ***	49.6807 ***	62.8730 ***	3.9253 ***	63.6275 ***
Stage × Cultivar (S × C)	7	3.4184 **	2.2034 *	3.4184 **	5.6438 ***	4.8394 ***	63.1775 *	3.6649 **
Stage × organ (S × T)	3	8.8900 ***	6.9457 **	8.8900 ***	16.6708 ***	NS	10.6520 ***	8.2789 ***
Cultivar × organ (C × T)	21	NS	NS	NS	NS	1.8119 *	NS	NS
Stage × Cultivar × organ (S × C × T)	21	NS	NS	NS	NS	NS	NS	NS

Figures in the figure represent F-values of different indicators. *: *p* < 0.05; **: *p* < 0.01; ***: *p* < 0.001. NS: insignificant difference.

**Table 4:** Enzyme contents determination methods link.

Name	Method Linkage
MDA	http://www.cominbio.com/a/shijihe/shenghuashiji/yanghuayukangyanghuaxilie/2018/0708/109.html (accessed on 15 March 2023)
GSSH	http://www.cominbio.com/a/shijihe/shenghuashiji/guguanggantaixilie/2014/1111/428.html (accessed on 15 March 2023)
GSH	http://www.cominbio.com/a/shijihe/shenghuashiji/guguanggantaixilie/2014/1111/427.html (accessed on 15 March 2023)
POD	http://www.cominbio.com/a/shijihe/shenghuashiji/yanghuayukangyanghuaxilie/2018/0708/153.html (accessed on 15 March 2023)
CAT	http://www.cominbio.com/a/shijihe/shenghuashiji/yanghuayukangyanghuaxilie/2019/0703/1843.html (accessed on 15 March 2023)
SOD	http://www.cominbio.com/a/shijihe/shenghuashiji/yanghuayukangyanghuaxilie/2018/0708/155.html (accessed on 15 March 2023)

## Data Availability

Data are contained within the article.

## Supplementary Materials

The following supporting information can be downloaded at: https://www.mdpi.com/article/10.3390/ijms24098019/s1.
