# Supplementary material for: Cadmium Absorption in Various Genotypes of Rice under Cadmium Stress
Source: Int J Mol Sci. 2023 Apr 28;24(9):8019. doi: 10.3390/ijms24098019 (PMC10178317; doi:10.3390/ijms24098019)
Supplement: Supplementary file 1 [file ijms-24-08019-s001.zip › ijms-2329308-supplementary.pdf]

Table S1. Total cadmium content of japonica rice cultivars in the first year of field

ID	*Japonica* rice varieties	Total cadmium content（mg·kg^{-1}）	ID	*Japonica* rice varieties	Total cadmium content（mg·kg^{-1}）
1	HJ753	0.0878±0.0019mn	25	WJN1116	0.1397±0.0009h
2	ZN14	0.0423±0.0004stuvw	26	NXJ-9	0.0903±0.0013lm
3	WYN4819	0.0808±0.007mn	27	YJ-13138	0.0755±0.0012mnop
4	HZXR-1	0.0601±0.0016pqrs	28	NJ58	0.0877±0.0009mn
5	TN2072	0.0777±0.0005mno	29	WXJ113	0.0922±0.0023klm
6	NJ2728	0.0596±0.0013pqrst	30	QXN-3	0.1109±0.0003jk
7	XD-9	0.0523±0.0014rstu	31	BHRM	0.0692±0.0049nopqr
8	HJ753	0.0794±0.0005de	32	HJ-5	0.1514±0.007gh
9	CYJ-7	0.0266±0.0004vw	33	YY4949	0.3720±0.004a
10	CLJ-1	0.0754±0.0011mnop	34	NJ5718	0.1735±0.003ef
11	NJ-3	0.0561±0.0009pqrstu	35	NJ46	0.0763±0.0009mno
12	TLY619	0.0242±0.0007w	36	FJ1606	0.2039±0.0019cd
13	WYJ245	0.0784±0.0008mno	37	WKN3 号	0.1605±0.01fg
14	ZD11	0.0544±0.0018qrstu	38	SXJ100	0.0739±0.0051mnopq
15	FJ908	0.0275±0.0009vw	39	S4699	0.2431±0.0018b
16	HD-5	0.0456±0.0005stv	40	WYN180	0.1537±0.0035fgh
17	NJ5055	0.0286±0.0041vw	41	ZD19	0.1531±0.0012gh
18	TXJ1402	0.1177±0.0061ij	42	WYJ3 号	0.1505±0.0022gh
19	SXJ1018	0.0774±0.0035mno	43	NJ-7	0.1392±0.0054h
20	SD1826	0.0395±0.0011uvw	44	WXJ109	0.1484±0.0109gh
21	YY4901	0.1356±0.0022hi	45	X63646	0.0840±0.0011mn
22	ZD29	0.0251±0.0032w	46	ZD18	0.1087±0.0028jkl
23	WKJ11036	0.0265±0.0038vw	47	SD301	0.1547±0.0005fgh
24	YY7826	0.0399±0.0007tuvw	48	ZXJ-1	0.2181±0.0014c

Table S2. Total cadmium content of indica rice cultivars in the first year of field

ID	*Indica* rice varieties	Total cadmium content（mg·kg-1）	ID	*Indica* rice varieties	Total cadmium content（mg·kg-1）
1	JHY7245	0.1671±0.0026h	25	JHY-7	0.2236±0.0004c
2	JHY-8	0.2369±0.0008d	26	WXY111	0.2302±0.0011dc
3	YHSM	0.1409±0.0007jk	27	NJZ	0.2059±0.0014d
4	JHY1586	0.3790±0.0011a	28	LY720	0.1356±0.001kl
5	TYXD	0.1303±0.0025lm	29	TFY-2	0.1777±0.0035fg
6	JXSZ	0.1717±0.0042gh	30	JHY-5	0.0859±0.0007tu
7	QLY155	0.3378±0.0036a	31	JLYJ-2	0.0961±0.0013rs
8	FLY1252	0.2864±0.0023b	32	XLY271	0.1790±0.0029fg
9	YLY2152	0.0806±0.0019uv	33	PY5627	0.2884±0.0009b
10	LLYHLZ	0.2493±0.0034c	34	YLY896	0.1798±0.0023fg
11	NX21	0.1077±0.0016pq	35	NLY-6	0.1213±0.0014mn
12	MXZ-1	0.1017±0.0006qr	36	HLY898	0.1732±0.0014gh
13	YXYLSM	0.1407±0.0034jk	37	TYXZ	0.0834±0.0006u
14	LLY1206	0.1455±0.0071ij	38	HZY261	0.1299±0.0004lm
15	JYYHSM	0.1143±0.002op	39	FLY-4	0.1516±0.0042i
16	TLY1413	0.1431±0.0037jk	40	JHY12	0.0934±0.0002st
17	YXYHS	0.1766±0.0016g	41	EFSM	0.1214±0.0016no
18	CLYHZ	0.1856±0.0027ef	42	HZY08	0.1144±0.0008op
19	JHY1583	0.2513±0.009c	43	LY73	0.1121±0.0014p
20	CY-9	0.0979±0.0042rs	44	MFSM	0.1774±0.0031g
21	TFY208	0.0922±0.0037st	45	MXZ-2	0.0568±0.0015w
22	W153	0.1464±0.0051ij	46	SYYSM	0.1487±0.0015ij
23	HHZ	0.1905±0.0014e	47	IIY838	0.0748±0.0024v
24	QY801	0.1769±0.0014g	48	G8YXSM	0.0351±0.0006x

Table S3. Total cadmium content of japonica rice cultivars in the second year of field

ID	*Japonica* rice varieties	Total cadmium content（$mg \cdot kg^{-1}$）	ID	*Japonica* rice varieties	Total cadmium content（$mg \cdot kg^{-1}$）
1	HJ753	0.2686±0.0055m	25	WJN1116	0.3432±0.0026jk
2	ZN14	0.1172±0.0049stuv	26	NXJ-9	0.3630±0.0063ij
3	WYN4819	0.0920±0.0021uvw	27	YJ-13138	0.1336±0.0032rstu
4	HZXR-1	0.1806±0.0009opq	28	NJ58	0.2046±0.0031no
5	TN2072	0.0730±0.0041vw	29	WXJ113	0.1283±0.0032rstu
6	NJ2728	0.1296±0.0040rstu	30	QXN-3	0.1441±0.0023qrst
7	XD-9	0.1456±0.0029qrs	31	BHRM	0.1489±0.0043qrs
8	HJ753	0.3171±0.0026kl	32	HJ-5	0.5404±0.0029bc
9	CYJ-7	0.3890±0.0078hi	33	YY4949	0.4205±0.0068fgh
10	CLJ-1	0.4550±0.0048ef	34	NJ5718	0.3814±0.0093hij
11	NJ-3	0.4366±0.0101fg	35	NJ46	0.6369±0.0354a
12	TLY619	0.6398±0.0106a	36	FJ1606	0.6541±0.0118a
13	WYJ245	0.3372±0.0103jk	37	WKN3 号	0.2514±0.0110m
14	ZD11	0.5704±0.0080b	38	SXJ100	0.1972±0.0013nop
15	FJ908	0.1567±0.0050pqrs	39	S4699	0.1599±0.004opqrs
16	HD-5	0.5471±0.0405bc	40	WYN180	0.3580±0.0086ijk
17	NJ5055	0.2328±0.01mn	41	ZD19	0.1671±0.0063opqr
18	TXJ1402	0.1721±0.0012opqr	42	WYJ3 号	0.0951±0.0042uvw
19	SXJ1018	0.1722±0.0037opqr	43	NJ-7	0.1474±0.0025qrs
20	SD1826	0.0948±0.0048uvw	44	WXJ109	0.2743±0.0137lm
21	YY4901	0.4016±0.0114ghi	45	X63646	0.0558±0.0013w
22	ZD29	0.1281±0.0016rstu	46	ZD18	0.5212±0.033dcd
23	WKJ11036	0.0993±0.0070tuvw	47	SD301	0.2731±0.0056opqr
24	YY7826	0.1197±0.051stu	48	ZXJ-1	0.4885±0.0064de

Table S4. Total cadmium content of indica rice cultivars in the second year of field

ID	*Indica* rice varieties	Total cadmium content（mg·kg-1）	ID	*Indica* rice varieties	Total cadmium content（mg·kg-1）
1	JHY7245	0.7087±0.0069a	25	JHY-7	0.5310±0.0009c
2	JHY-8	0.6956±0.0047a	26	WXY111	0.2129±0.0019no
3	YHSM	0.2327±0.0016m	27	NJZ	0.1809±0.0007pq
4	JHY1586	0.4891±0.0027d	28	LY720	0.1532±0.0041tuv
5	TYXD	0.1790±0.0085pqr	29	TFY-2	0.3126±0.0036k
6	JXSZ	0.3509±0.0069ij	30	JHY-5	0.6493±0.006b
7	QLY155	0.4530±0.0019ef	31	JLYJ-2	0.2723±0.0066l
8	FLY1252	0.3275±0.0075k	32	XLY271	0.5255±0.0024c
9	YLY2152	0.2802±0.0056l	33	PY5627	0.4308±0.0012g
10	LLYHLZ	0.6666±0.0012b	34	YLY896	0.2717±0.0068l
11	NX21	0.1725±0.0106qrst	35	NLY-6	0.1591±0.0007stuv
12	MXZ-1	0.1484±0.0003uv	36	HLY898	0.1510±0.0036uv
13	YXYLSM	0.5145±0.0285c	37	TYXZ	0.1169±0.0024x
14	LLY1206	0.4377±0.0051fg	38	HZY261	0.1649±0.0012qrstu
15	JYYHSM	0.3793±0.0059h	39	FLY-4	0.2282±0.0013mn
16	TLY1413	0.3578±0.0057i	40	JHY12	0.1736±0.0070qrs
17	YXYHS	0.2283±0.0076mn	41	EFSM	0.2228±0.0044mn
18	CLYHZ	0.1409±0.0003vw	42	HZY08	0.1978±0.0026op
19	JHY1583	0.4628±0.048e	43	LY73	0.1595±0.0014rstuv
20	CY-9	0.1287± 0.0053wx	44	MFSM	0.2400±0.0016m
21	TFY208	0.0946±0.0003y	45	MXZ-2	0.1112±0.0028xy
22	W153	0.1663±0.0019qrstu	46	SYYSM	0.1623±0.0002qrstu
23	HHZ	0.1541±0.0030stuv	47	IIY838	0.3319±0.0055jk
24	QY801	0.3866±0.0021h	48	G8YXSM	0.0948±0.0072y

Table S5. Names of genes associated with cadmium uptake in rice

ID	Primer	Primer sequences（5′to 3′）
1	OsHMA3 F	CGACTCCCAAGCACCCAACG
2	OsHMA3 R	GGCAGCCGTTCGTTTGCT
3	LCD F	ACCCAAACTTATGATTCTAC
4	LCD R	CAATCATCAGGGTAGTTT
5	CAL1 F	GCGTCCGCCTTCCTCCTC
6	CAL1 R	TTGCACTCGCCGTCGGGGAAGC
7	OslRT1 F	GACACTGGTGCCCATTCTGCC
8	OslRT1 R	GGCGAGGTGAGGTTGTTGAAGG
9	OslRT2 F	CGCAGCTTCTCAGGAATCGC
10	OslRT2 R	GCGTCGTGGTGGAGAAGAAGA
11	OsNRAMP1 F	GTAATGCCGCACAATCTA
12	OsNRAMP1 R	TGCTGATGCGGGTGTATT
13	OsNRAMP5 F	CAGAGGAATCAAGGACGGGTGC
14	OsNRAMP5 R	CCTGATGTCCAAGAAACCCT
15	OsMTP1 F	CAGCCCATCTCCTTTCGG
16	OsMTP1 R	TAGAAACCAGGGCACCAA
17	OsPCR1 F	TGTATGGCGTGCTGGTGC
18	OsPCR1 R	CAGCAGAAGGTGACGAGGC
19	OsCCX2 F	TCGTGTCCACCGTTGTTGCTG
20	OsCCX2 R	GCGGATAGGAGGCAATGAAGGT
21	OsLCT1 F	CTTTGCTGCGTTCTACCG
22	OsLCT1 R	CAGGACCTTCATCCACAGCA
23	OsHMA9 F	ACGGCATCAACGACTCCC
24	OsHMA9 R	ACGCTGACGGAGGAGAACG
